# Editorial: Precision medicine for antithrombotic therapy in patients after percutaneous coronary interventions

**DOI:** 10.3389/fcvm.2023.1162630

**Published:** 2023-03-03

**Authors:** Mattia Galli, Francesco Costa, Dominick J. Angiolillo

**Affiliations:** ^1^ Department of Cardiology, Maria Cecilia Hospital, GVM Care & Research, Cotignola, Italy; ^2^ Department of Biomedical and Dental Sciences and Morphological and Functional Imaging, University of Messina, Messina, Italy; ^3^ Division of Cardiology, University of Florida College of Medicine, Jacksonville, FL, United States

**Keywords:** precision medecine, antithrombic agent, antiplatelet therapy, percutaneous coronary intervention, anticoagulant, P2Y12 inhibitors

**Editorial on the Research Topic** Precision medicine for antithrombotic therapy in patients after percutaneous coronary interventions

Precision medicine is an innovative medical model aiming at providing the right treatment to the right patient at the right time (1). After percutaneous coronary interventions (PCI), antiplatelet therapy plays a key role in preventing thrombotic events such as stent thrombosis or myocardial infarction, but is inevitably associated with increased bleeding (2). Given the inter-patient variability in response to antiplatelet therapy, a multi-factorial approach that accounts for clinical, procedural, and genetic factors is necessary for attaining an optimal balance between ischemic and hemorrhagic events. A precise, individualized, strategy is needed as opposed to a standard “one-size-fits all” approach (1). In this Special Issue of Frontiers in Cardiovascular Medicine, the authors provide relevant evidence on the impact of different antiplatelet treatment regimens on outcomes and on the different response that these regimens may display in specific subgroup of patients.

Antiplatelet therapy may be either intensified/prolonged or de-escalated/shortened to achieve an optimal trade-off between ischemic and bleeding events according to the individual patient characteristics (2, 3). In their original contribution, Kwan Young Lee et al. sought to explore the effectiveness of extended (>24 months) dual antiplatelet therapy (DAPT) in high ischemic risk acute coronary syndrome (ACS) patients undergoing PCI who had no major bleeding after at least 1 year of DAPT. Ischemic risk was defined according to the PEGASUS-TIMI 54 criteria. They found extended DAPT was associated with a lower risk of mortality without increasing the risk of major bleeding among 2 years survivors after ACS who met the PEGASUS criteria and had no major bleeding events before 24 months. Similar results were also observed in the report from the Korean nationwide registry including 273,670 Korean PCI patients by Seung-Jung Lee et al. that suggests prolonged (1–3 years) DAPT may be particularly beneficial in diabetic patients. The further contribution by Ana Lucrecia Marcano et al. explore the pathophysiological mechanisms at the basis of these clinical findings in diabetes mellitus (DM) patients. In their pharmacodynamic, crossover, study randomizing Mediterranean patients with DM to either ticagrelor (*n* = 20) or clopidogrel (*n* = 20), the authors found that ticagrelor was associated with greater platelet inhibition after a loading dose and at 1 week, compared with clopidogrel. These finding are in line with previous reports (4). Collectively, evidence from these studies support an intensified/prolonged antithrombotic regimen may be particularly useful in high-ischemic risk patients, particularly if not at high risk for bleeding (5).

The ever growing understanding of the prognostic impact of bleeding events, the availability of less thrombogenic stent platforms and the notion that the ischemic risk is highest during the first 1–3 months after PCI/ACS, have fueled interest in implementing the so-called “de-escalation” strategies (6, 7). A comprehensive appraisal of these strategies is provided in the review article by Marie Muthspiel et al. while the network meta-analysis by Oumaima El Alaoui El Abdallaoui et al. allows for a direct and indirect comparison between different de-escalation strategies among 42,511 patients from 10 randomized controlled trials (RCTs). Their findings suggest both a strategy of de-escalation of P2Y_12_ inhibitor intensity and a strategy of P2Y_12_ inhibitor monotherapy may be associated with better outcomes compared to standard DAPT among ACS patients undergoing PCI. Moreover, they speculate that the former strategy may be more effective in reducing ischemic events while the latter strategy may be more effective in reducing bleeding, compared to standard DAPT.

The individual response to specific antiplatelet agents may be affected by clinical variables but also by sex-related, genetic, and demographics characteristics (1). The increasing awareness of the different response to antithrombotic agents woman may exhibit as opposed to men has been subject of growing interest (8). Indeed, females are often underrepresented in RCTs and the so called “Yentl syndrome” identifies the issues related to the paucity of evidence focusing on the subgroup of woman (8). To this extent, Laborante et al. provide a comprehensive summary of the evidence on gender-differences in antithrombotic therapy in ischemic heart disease, discussing the future perspectives for tackling the Yentl syndrome. Demographic characteristics, especially those concerning the different ischemic and bleeding risks across Asian versus Non-Asian patients, are one of the leading confounding factors in the appraisal of RCTs on antithrombotic therapy (1). Indeed, Asian patients display an increased risk of bleeding and a reduced risk of ischemic events compared with non-Asian patients, limiting the application of the evidence from RCTs between these populations (9). The response to specific P2Y_12_ inhibitors, in particular clopidogrel, may be predicted according to the genotype responsible for the transcription of the enzyme that leads to clopidogrel metabolism, the cytochrome (CYP) C219 (10). In fact, the presence of CYP2C19 “loss-of-function” (LoF) alleles is associated with reduced generation of clopidogrel's active metabolite, high platelet reactivity and increased rates of thrombotic complications (11). However, the prevalence of CYP2C19 LoF alleles is significantly affected by ethnicity (10). Interestingly, Asian patients present increased bleeding and lower ischemic risks compared with other ethnicities despite the higher prevalence of CYP2C19 LoF alleles compared to the general population, contributing to the so called “Asian Paradox” (9). Studies like that by Yu-Wei Chen et al. exploring the impact on outcomes of CYP2C19 LoF in 999 East Asian patients with ACS undergoing PCI, have the important role of supporting the clinical impact of this genetic phenotype across difference ethnicities, despite the inherent differences in bleeding and ischemic risks between these populations (9). To this extent, it is of great interest the review article from Anh B Nguyen et al. discussing the race and ethnicity disparities in outcome studies of CYP2C19 genotype-guided antiplatelet therapy.

Finally, the management of antithrombotic therapy in patients with atrial fibrillation and concomitant ACS or PCI still represents a clinical conundrum (12). In this setting, it is of utmost importance the adequate selection of patients that may benefit the most from different antithrombotic regimens, given that the association between anticoagulants and antiplatelet leads to a particularly high risk of bleeding (13, 14). The study by Zhitong Li et al. adds important information on the additive role of atrial cardiomyopathy, assessed by B-type natriuretic peptide, P-wave terminal force in ECG lead V1, and left atrium diameter, on top of the standard CHA_2_DS_2_-VASc score for the prediction of the risk of cerebrovascular events in ACS patients.
